# Hypertension Subtypes and Diabetes: Joint Associations in the National Health and Nutrition Examination Survey

**DOI:** 10.1016/j.jacadv.2026.102603

**Published:** 2026-02-24

**Authors:** Jeffrey E. Jones, Kevin S. Tang, James C. Franklin, Wenjun Fan, Nathan D. Wong

**Affiliations:** a Heart Disease Prevention Program, Mary and Steve Wen Cardiovascular Division, Department of Medicine, University of California, Irvine, California, USA; b Department of Epidemiology and Biostatistics, University of California, Irvine, California, USA

**Keywords:** cardiovascular disease, diabetes mellitus, hypertension

## Abstract

**Background:**

Diabetes mellitus (DM) is associated with increased arterial stiffness indicated by isolated systolic hypertension (ISH). Whether the prevalence of ISH and other hypertension (HTN) subtypes differs by DM status and how they relate to outcomes remains unclear.

**Objectives:**

The objective of the study was to compare by DM status the association of HTN subtypes with cardiovascular disease (CVD) and all-cause mortality.

**Methods:**

The authors included data from the National Health and Nutrition Examination Survey with mortality follow-up until 2019. The authors used HTN thresholds of 130/80 mm Hg to define ISH, isolated diastolic hypertension (IDH), and systolic-diastolic hypertension (SDH). The incidence of CVD and all-cause mortality was examined among hypertensive subtypes using multivariable Cox regression.

**Results:**

A total of 52,385 individuals (projected to 207 million) were included, of which 12% (25 M) had DM. In those with vs without DM, 23% vs 57% were normotensive, 5% vs 9% had IDH, 39% vs 18% had ISH, and 16% vs 13% had SDH (all *P* < 0.01). Analyses adjusted for age, sex, and other risk factors showed significantly increased risks for CVD and all-cause mortality with ISH (HR: 1.19; *P* < 0.01 and HR: 1.15; *P* < 0.01, respectively) and SDH (HR: 1.29; *P* < 0.01 and HR: 1.18; *P* < 0.01) but not IDH in patients without DM (HR: 0.76; *P* = 0.32 and HR: 0.86; *P* = 0.20); in patients with DM, this risk was increased in patients with ISH (HR: 1.57; *P* < 0.01 and HR: 1.46; *P* < 0.01], SDH (HR: 2.11; *P* < 0.01 and HR: 1.68; *P* < 0.01), and IDH (HR: 3.65; *P* < 0.01 and HR: 1.97; *P* < 0.01) (interaction *P* < 0.01 for all subtypes by DM status).

**Conclusions:**

HTN subtypes were significantly associated with increased CVD and all-cause mortality in patients with DM more strongly than in those without DM, suggesting greater importance of controlling all HTN subtypes in those with DM.

Diabetes mellitus (DM) is a global health challenge and a major contributor to cardiovascular disease (CVD) morbidity and mortality.^1^, ^2^, ^3^ Individuals with DM face a significantly higher risk of cardiac, neurologic, and renal disease, leading to 2 to 4 times greater CVD risk compared to the general population.^4^ Early identification and treatment of DM and its sequelae have been shown to reduce the risk for major adverse CVD events.^4^ Hypertension (HTN) often accompanies DM, as both conditions are associated with vascular injury and arterial stiffness.^5^^,^^6^ Both HTN and DM are independently associated with an increased risk for CVD, and adequate blood pressure and glucose control can reduce excess CVD risks in at-risk populations.^4^^,^^7^

Various studies have shown a significant association between elevated systolic blood pressure (SBP) and CVD risk.^8^^,^^9^ However, the CVD risk associated with diastolic HTN, a condition mainly found in young individuals, has not been well defined.^10^^,^^11^ Early work by the Framingham Heart Study and others identified diastolic HTN among the risk factors for coronary heart disease.^12^, ^13^, ^14^ Later studies, including the New York Work-Site Hypertension Control Program, the Honolulu Heart Program, the Copenhagen City Heart Study, and the Finnish Male Cohort Study, characterized isolated diastolic hypertension (IDH) as a benign condition without increased mortality risk in the general population.^15^, ^16^, ^17^, ^18^ Although DM has been identified as an independent risk factor for the development of both HTN and CVD,^5^ the implication of DM on mortality risk among HTN subtypes has not been well characterized. The current study aims to assess the prevalence of HTN subtypes among U.S. adults with and without DM and to evaluate the comparative relations of HTN subtypes on CVD and all-cause mortality.

## Methods

### Data source

The National Health and Nutrition Examination Survey (NHANES) is a nationally representative cross-sectional survey administered by the Centers for Disease Control and Prevention. Each year since 1999, the survey has interviewed ∼5,000 noninstitutionalized individuals in counties across the country, of which 15 are visited in person. Variables collected included patient demographics, physical exams, serologic data, and health questionnaires. Blood pressure was collected by trained physicians in 30 second intervals following 5 minutes of rest in a seated position. Three readings were collected from each individual, with a fourth reading collected if any of the initial 3 readings were unobtainable. We included U.S. adults aged 20 or older with valid blood pressure measurements from NHANES 1999 to 2020 for the analysis of the prevalence of HTN subtypes by diabetes status. The mean blood pressure was calculated from the recorded SBP and diastolic blood pressure (DBP) values. In addition, mortality data from the National Death Index was linked to NHANES and classified by International Classification of Diseases-10 codes. For mortality analyses, we defined a baseline cohort from 1999 to 2008 with follow-up to 2019. This study used deidentified publicly available data and thus was exempt from Institutional Review Board review.

### Definitions

HTN was defined as elevated systolic (≥130 mm Hg) or diastolic (≥80 mm Hg) blood pressure per the 2017 and 2025 American College of Cardiology/American Heart Association Blood Pressure Guidelines.^19^^,^^20^ An SBP ≥130 mm Hg and DBP <80 mm Hg was defined as isolated systolic hypertension (ISH). Similarly, an SBP <130 mm Hg with DBP ≥80 mm Hg was defined as IDH. Systolic-diastolic hypertension (SDH) was defined as elevated SBP and elevated DBP. Separate sensitivity analyses were conducted using the 7th Report of the Joint National Committee definitions of HTN using SBP and DBP cutoffs of 140 mm Hg and 90 mm Hg, respectively.^21^ Patient age, sex, race, ethnicity, educational attainment, smoking history, and the use of antihypertensive medication were obtained via patient report. Prior CVD was defined by participant report of being diagnosed with coronary heart disease, angina pectoris, heart attack, or stroke. Body mass index was calculated from a digital weight scale and standing height. Patients with DM were identified by fasting glucose ≥126 mg/dL, nonfasting glucose ≥200 mg/dL, hemoglobin A1C ≥ 6.5%, currently taking insulin or diabetic medication, or having been told by a physician that they have DM. Dyslipidemia was defined as total cholesterol ≥200 mg/dL, low-density lipoprotein ≥130 mg/dL, high-density lipoprotein <35 mg/dL, or triglycerides ≥150 mg/dL. Obesity was defined by body mass index ≥30 kg/m^2^. CVD death was defined as death from heart (International Classification of Diseases–10th Revision codes I00-I09, I11, I13, I20-I51) or cerebrovascular disease (I60-169).^22^

### Statistical analysis

Each 2-year NHANES cohort was weighted to produce nationally representative data for the adult noninstitutionalized United States population by dividing each sample weight by the number of survey cycles combined.^23^ Continuous variables are presented as mean (SE), and categorical variables as unweighted n (with sample weighting used for calculation of weighted N and weighted %). Categorical group differences were assessed using the chi-square test. For the survival analysis, the risk of CVD and all-cause mortality were compared between individuals with and without DM according to HTN subtype (with the reference group being those with normal or controlled blood pressure) using Cox proportional hazards regression, adjusting for age, race/ethnicity, sex, educational attainment (as a measure of social determinant of health), obesity, tobacco use, dyslipidemia, the use of antihypertensive pharmacotherapy, and prior CVD. Proportional hazard assumptions were checked via Kaplan-Meier curves showing HTN subgroups to be concordant and without significant crossovers. HRs are presented alongside 95% CIs with interaction terms tested between those with and without DM. Statistical analyses were performed with SAS statistical software (version 9.4; SAS Institute).

## Results

### Study sample demographics and hypertension subtypes

**Figure 1:**
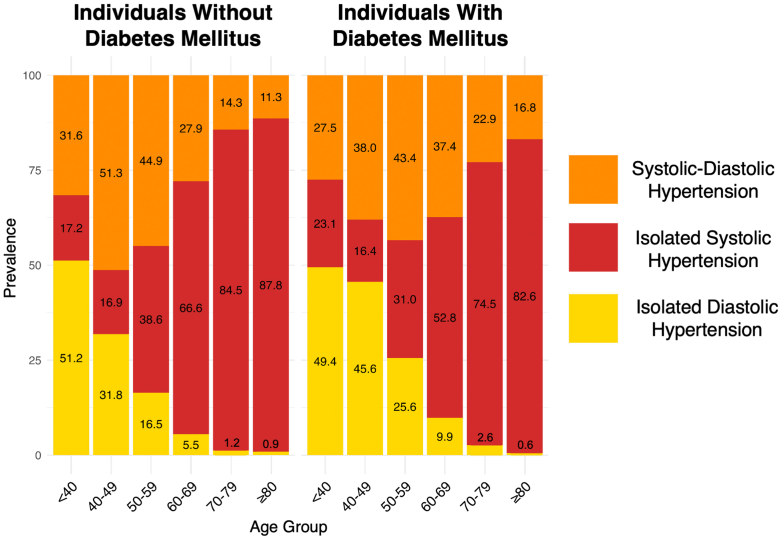
Prevalence (%) of Hypertension Subtypes by Age in U.S. Adults With and Without Diabetes Mellitus

A total of 58,744 adults were surveyed by the NHANES from 1999 to 2020, of which 52,385 (projected to 207 million) had valid blood pressure measurements. Of these, 21,531 were defined to have hypertension, including 4,900 (representing 13 million) with diabetes and 16,631 (representing 63 million) (Central Illustration). Table 1 shows descriptive statistics of our study sample comparing those with vs without DM, and Supplemental Table 1 compares baseline characteristics between included and excluded participants. Overall, 12% of the study population met the criteria for DM, projected to 25 million individuals. Patients with DM were more likely to have HTN (77.8%) compared to those without (43.2%, *P* < 0.01). Prevalence of HTN subtypes was 56.2% ISH, 13.3% IDH, and 30.5% SDH in adults with DM compared to 37.8% ISH, 28.6% IDH, and 33.6% SDH in adults without DM (*P* < 0.01). When using the older cutoffs of 140 mm Hg for SBP and 90 mm Hg for DBP, supplementary analysis showed prevalence of 76.4% ISH, 8.2% IDH, and 15.4% SDH in adults with DM compared to 65.3% ISH, 14.3% IDH, and 20.3% SDH in adults without DM. Other comparisons between HTN patients with and without DM are shown in Table 1. IDH was the most prevalent HTN subtype in younger age groups and became less prevalent with increasing age. In those with and without DM, greater percentages of ISH were observed to increase in prevalence with age, becoming the most prevalent HTN subtype in individuals above the age of 50 (Figure 1). Greater age was associated with higher prevalence of ISH and SDH in both individuals with and without DM (*P* < 0.01) (Figure 1).

**Central Illustration:**
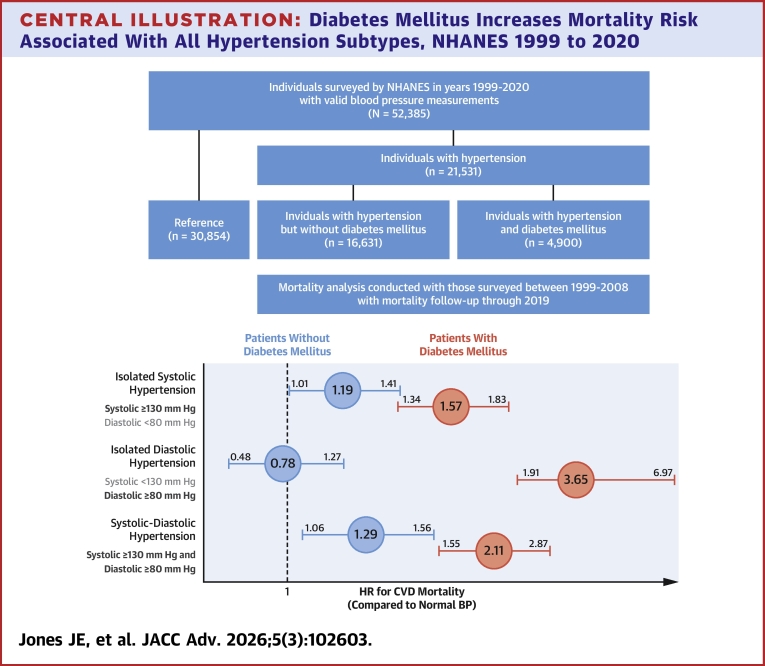
**Diabetes Mellitus Increases Mortality Risk Associated With All Hypertension Subtypes, NHANES 1999 to 2020** BP = blood pressure; CVD = cardiovascular disease; NHANES = National Health and Nutrition Examination Survey.

**Table 1:** Demographic Characteristics and Comorbidities Among U.S. Adults With Hypertension, NHANES 1999-2020

	Individuals Without DM	Individuals With DM	*P* Value
Total (N = 21,531)	16,631 (63 M)	4,900 (13 M)	<0.01
Mean age	45.27 years (0.2)	59.25 years (0.3)	<0.01
Female	7,662 (29 M, 46.5%)	2,356 (6.5 M, 48.6%)	0.07
Isolated systolic hypertension	7,145 (24 M, 37.8%)	2,988 (7.6 M, 56.2%)	<0.01
Isolated diastolic hypertension	3,790 (18 M, 28.6%)	496 (1.8 M, 13.3%)	
Systolic-diastolic hypertension	5,696 (21 M, 33.6%)	1,416 (4.1 M, 30.5%)	
On antihypertensive therapy	9,147 (35 M, 56.3%)	2,131 (6.4 M, 49.2%)	0.02
Ethnicity			
Non-Hispanic Black	4,085 (8.0 M, 12.6%)	1,441 (2.3 M, 17.2%)	<0.01
Hispanic	3,443 (6.6 M, 10.4%)	1,380 (2.0 M, 14.8%)	
Non-Hispanic White	7,705 (45 M, 70.5%)	1,665 (8.1 M, 59.9%)	
Other	1,398 (4.1 M, 6.5%)	404 (1.1 M, 8.1%)	
Educational attainment			
<9th grade	1,986 (3.6 M, 5.7%)	926 (1.4 M, 10.7%)	<0.01
9th-11th grade	2,445 (7.3 M, 11.5%)	868 (1.9 M, 14.4%)	
High school graduate or equivalent	4,137 (17 M, 26.2%)	1,168 (3.6 M, 27.4%)	
Some college or associates degree	4,664 (19 M, 30.6%)	1,247 (3.9 M, 29.8%)	
College graduate or above	3,380 (16 M, 25.9%)	681 (2.3 M, 17.7%)	
Smoking history			
Never smoking	24,242 (99 M, 54.6%)	1,443 (12 M, 49.0%)	<0.01
Former smoking	10,034 (43 M, 23.6%)	2,865 (8.6 M, 34.2%)	
Current smoking	9,430 (40 M, 21.8%)	1,443 (4.2 M, 16.8%)	
Obesity			
BMI 18.5-24.9	4,081 (15 M, 24.5%)	642 (1.5 M, 11.7%)	<0.01
BMI 25-29.9	5,726 (22 M, 35.3%)	1,420 (3.5 M, 26.7%)	
BMI ≥30	6,320 (25 M, 40.2%)	2,672 (8.1 M, 61.6%)	
Dyslipidemia	8,989 (36 M, 56.3%)	2,354 (6.6 M, 49.2%)	<0.01
Total cholesterol (mg/dL)	187.76 (0.5)	180.36 (0.95)	<0.01
High-density lipoprotein cholesterol (mg/dL)	51.61 (0.3)	45.29 (0.5)	<0.01
Triglycerides (mg/dL)	124.56 (0.1)	173.11 (2.8)	<0.01
Prior cardiovascular disease	1,780 (5.4 M, 8.5%)	1,016 (2.5 M, 19.0%)	<0.01

Prevalence of demographic characteristics, hypertension subtypes, and total lipid levels among U.S. adults with and without diabetes mellitus, NHANES 1999 to 2020. ISH (SBP ≥130 mm Hg and DBP <80 mm Hg), IDH (DBP ≥80 mm Hg and SBP <130 mm Hg), and SDH (SBP ≥130 mm Hg and DBP ≥80 mm Hg).

BMI = body mass index; DM = diabetes mellitus; NHANES = National Health and Nutrition Examination Survey.

### Mortality trends among patients with diabetes mellitus

**Figure 2:**
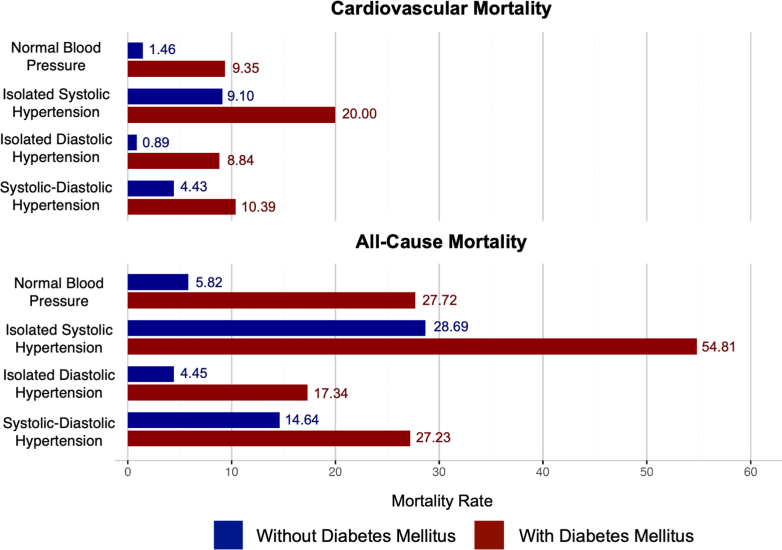
Unadjusted Mortality Rates per 1,000 Years for Hypertension Subtypes According to Diabetes Status

**Figure 3:**
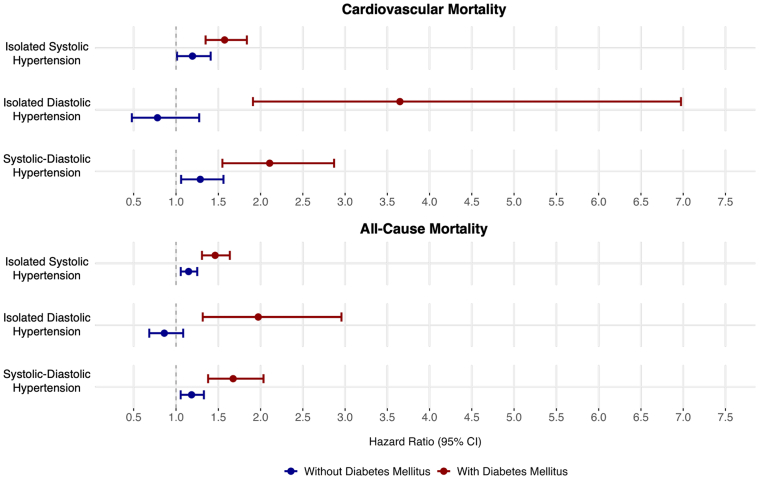
**HRs for the Mortality Risk by Hypertension Subtype and Diabetes Status Relative to Those With Normotension** Analyses adjusted for age, sex, ethnicity, obesity, tobacco use, dyslipidemia, and use of antihypertensive agents.

After an average follow-up of 14.6 years, individuals with DM faced higher mortality risk across all HTN subtypes compared to those without DM (Figure 2 and Central Illustration). In unadjusted analyses, individuals with ISH had the highest CVD and all-cause mortality risk, followed by SDH, normotension, and IDH. After adjusting for age, ethnicity, sex, educational attainment, obesity, tobacco use, dyslipidemia, the use of antihypertensive agents, and prior CVD, individuals with DM faced higher CVD mortality risk from ISH (HR: 1.57; *P* < 0.01 vs HR: 1.19; *P* < 0.01, *P* < 0.01 for interaction between DM and HTN subtypes) and from SDH (HR 2.11; *P* < 0.01 vs HR: 1.29; *P* < 0.01 for interaction) compared to those without DM. All-cause mortality analyses demonstrated similar trends for ISH (DM HR: 1.46; *P* < 0.01; non-DM HR: 1.15; *P* < 0.01, interaction *P* < 0.01) and SDH (DM HR: 1.68; *P* < 0.01; non-DM HR: 1.18; *P* < 0.01, interaction *P* < 0.01) compared to normotension. IDH in patients without DM was not associated with an increased risk of CVD (HR: 0.78; *P* = 0.32) or all-cause (HR: 0.86; *P* = 0.20) mortality. However, IDH in patients with DM had a strong association with CVD (HR: 3.65; *P* < 0.01) and all-cause mortality (HR: 1.97; *P* < 0.01) (interaction *P* < 0.01 for both compared to non-DM). Three-way interactions for HTN, DM, and age were significant at *P* < 0.01 for all comparisons (Table 2, Figure 3). When stratifying HTN subtypes according to age and DM status, ISH, and SDH were associated with increased CVD and all-cause mortality risk in all age groups (Supplemental Table 2). IDH was not associated with mortality in any age group without DM. In those with DM and IDH, individuals <65 years of age faced significant CVD (HR: 5.16; *P* < 0.01) and all-cause mortality (HR: 2.45; *P* < 0.01). Those ≥65 years of age did not experience increased mortality (CVD HR: 0.96; *P* = 0.97 and all-cause HR: 0.97; *P* = 0.94).

**Table 2:** Adjusted HRs for Cardiovascular and All-Cause Mortality by Hypertension Subtype for U.S. Adults With and Without Diabetes Mellitus, NHANES 1999–2020

	Cardiovascular Mortality		All-Cause Mortality	
	Without DM	With DM	Without DM	With DM
Age (per year)	1.10 (1.09-1.10)	1.12 (1.10-1.12)	1.08 (1.08-1.09)	1.09 (1.09-1.10)
Normotension	1.00	1.00	1.00	1.00
Isolated systolic hypertension	1.19 (1.01-1.41)	1.57 (1.34-1.83)	1.15 (1.06-1.25)	1.46 (1.31-1.63)
Isolated diastolic hypertension	0.78 (0.48-1.27)	3.65 (1.91-6.97)	0.86 (0.68-1.08)	1.97 (1.32-2.96)
Systolic-diastolic hypertension	1.29 (1.06-1.56)	2.11 (1.55-2.87)	1.18 (1.05-1.33)	1.68 (1.38-2.03)
HTN/DM interaction	*P* < 0.01		*P* < 0.01	
HTN/DM/age interaction	*P* < 0.01		*P* < 0.01	

All analyses adjusted for age, ethnicity, sex, educational attainment, obesity, tobacco use, dyslipidemia, use of antihypertensive agents, and prior cardiovascular disease.

HTN = hypertension; other abbreviations as in Table 1.

In a sensitivity analysis, when using the JNC7 cutpoints for HTN of 140/90 mm Hg, HTN subtype distributions across ages and mortality outcome trends were similar (Supplemental Table 3). ISH and SDH were significantly associated with increased CVD and all-cause mortality, whereas IDH was only associated with mortality in those with DM (Supplemental Table 3). Consistent results were observed in our sensitivity analysis excluding those on antihypertensive therapy.

## Discussion

In our analysis of over 52,000 individuals (projected to 207 million) surveyed by the NHANES from 1999 to 2020, ISH was the predominant HTN subtype both in those with and without DM, and in the presence of DM was associated with the highest absolute CVD and all-cause mortality rates. Importantly, all HTN subtypes were stronger predictors of CVD and all-cause mortality in those with DM compared to those without DM. To our knowledge, this is the first study to examine mortality risk differences among HTN subtypes in adults with DM.

The significant interaction between HTN and DM seen in both CVD and all-cause mortality indicates that DM compounds the mortality risk attributed to HTN. In our study, mortality risks associated with each HTN subtype were greater in individuals with DM compared to those without, and even those considered normotensive still faced a higher rate of CVD and all-cause mortality compared to normotensive individuals without DM. This is consistent with the broader pathophysiology of DM, which confers CVD risk through arterial stiffening and HTN, but has further been linked to progressive microvascular dysfunction and other complications.^24^^,^^25^ The three-way interaction among HTN, DM, and age further suggests that certain subtypes of HTN are stronger predictors of mortality in different age groups. Specifically, IDH was only associated with CVD and all-cause mortality in younger individuals with DM whereas ISH and SDH were significantly associated with mortality in all age groups and in those with and without DM. These data suggest that there is a role for DBP control in younger patients with DM, in addition to SBP and glycemic control for all patients with DM.

The association of IDH with increased CVD and all-cause mortality in those with DM in our analysis is also noteworthy. Diastolic HTN has long been considered a relatively benign variant of HTN, predominantly occurring in younger adults, with the majority of HTN mortality risk attributed to elevations in SBP.^6^^,^^10^^,^^26^ Recent data have begun to suggest some association between IDH and mortality, even in young individuals. A 2015 study of participants from the Chicago Heart Association Detection Project in Industry study with an average follow-up time of 31 years found a significant association between IDH and CVD mortality.^26^ These individuals with IDH were found to have higher peripheral vascular resistance compared to those with ISH, suggesting that individuals with IDH have early evidence of arterial stress that may later manifest as SDH or convert to ISH. A 2020 study using health screening data from the Korean National Health Insurance database with a median follow-up of 13.2 years found that individuals with IDH had an increased risk of CVD events compared to those with normal blood pressure and a comparable risk of CVD events as those with ISH and SBP 130 to 139 mm Hg.^27^ The mixed data regarding the mortality risk of IDH may be related to the follow-up time required to observe a difference in mortality, or the effect of IDH superimposed on other CVD risk factors like DM. Our results suggest that in younger patients with DM, clinicians should reserve special attention for DBP and consider treating diastolic HTN, even in patients with normal SBP.

### Study Limitations

Our analysis has several strengths and limitations. The NHANES is the most representative U.S. population survey which gives important implications for the greater U.S. population. Moreover, standardized protocols were used for the measurement of blood pressure as well as laboratory measures and ascertainment of medical history and comorbidities. However, NHANES cannot definitively differentiate between type 1 and type 2 DM. Given that type 1 DM accounts for the majority of diabetes cases in younger age groups,^28^^,^^29^ the same age group with high prevalence of IDH, the increased mortality risk seen in DM patients with IDH could reflect the longer duration of DM in these individuals. The treatment of young individuals with DM and IDH, who have a low baseline mortality risk, requires careful consideration. There also remains the potential for residual confounding by factors not appropriately evaluated by NHANES data collection methodology. This may include other socioeconomic factors, health literacy, changing HTN treatment guidelines, or potential birth cohort effects such as generational changes in lifestyle given the duration of follow-up. However it should be noted that prior NHANES analyses have indicated that the prevalence of healthy lifestyle behaviors have been relatively consistent across survey years.^30^ Finally, the results of our analysis are derived from U.S. adults and may not be generalizable to non-U.S. populations, although it should be noted that the prevalence trends reported here are consistent with other analyses of both U.S. and non-U.S. cohorts.

## Conclusions

Our study demonstrates a distinct distribution of HTN subtypes in patients with DM compared to those without, especially in younger age groups. Importantly, DM is associated with substantially higher mortality risks across all HTN subtypes. IDH, a subtype not typically associated with increased mortality in those without DM, exhibited a significant association with increased mortality, particularly in young patients with DM. These findings underscore the complex interplay between DM, specific HTN subtypes, and mortality risk. Further research is warranted to investigate the impact of other comorbidities on mortality risk within specific HTN subtypes. A comprehensive understanding of the interplay between cardiovascular comorbidities is crucial for optimizing CVD prevention and management strategies in patients with HTN.

Perspectives **COMPETENCY IN MEDICAL KNOWLEDGE:** Understanding how HTN subtypes confer different mortality risks in those with and without DM is necessary for optimal cardiovascular risk assessment.

**TRANSLATIONAL OUTLOOK:** Future research should investigate the cardiovascular and all-cause mortality risk of long-standing IDH in individuals with DM. Studies involving treatment of IDH could improve cardiovascular outcomes in specific patient populations, such as those with DM.

## Funding support and author disclosures

The study was supported in part by the Stanley S. Franklin MD Memorial Endowment Fund for Hypertension and Heart Disease Prevention Research. The authors have reported that they have no relationships relevant to the contents of this paper to disclose.

## Supplementary material

Supplementary data1
