# Supplementary material for: Hypertension Subtypes and Diabetes: Joint Associations in the National Health and Nutrition Examination Survey
Source: JACC Adv. 2026 Feb 24;5(3):102603. doi: 10.1016/j.jacadv.2026.102603 (PMC12955115; doi:10.1016/j.jacadv.2026.102603)
Supplement: Supplementary data1 [file mmc1.docx]

**Supplemental Appendix**

**Supplemental Figure 1. Hypertension subtype distribution by age group for US adults with and without diabetes mellitus using JNC7 hypertension cutoffs of 140/90 mmHg, NHANES 1999–2020.**

**
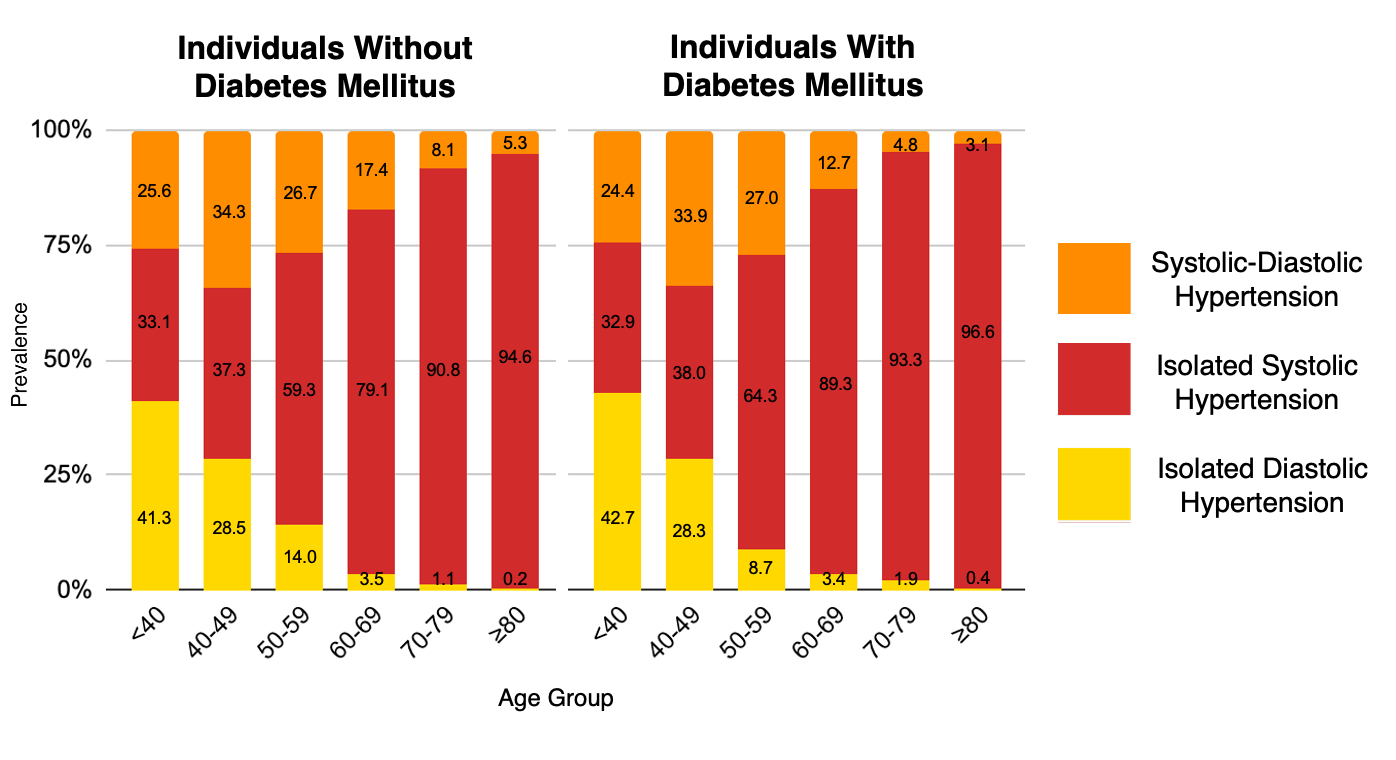
**

Prevalence (%) of hypertension subtypes by age in US adults with and without diabetes mellitus.

**Supplemental Table 1. Demographic characteristics and comorbidities among included and excluded individuals in the study, NHANES 1999-2020.**

|  | Included individuals | Excluded individuals |
| --- | --- | --- |
| Total (N=58,744) | 52,385 (207 M) | 6,359 (21 M) |
| Mean age | 46.97 years (0.2) | 47.15 years (0.5) |
| Female | 27,029 (107 M, 51.6%) | 3,748 (13 M, 58.9%) |
| Ethnicity | | |
| Non-Hispanic Black | 11,282 (23 M, 11.1%) | 1,619 (3.3 M, 15.5%) |
| Hispanic | 13,294 (29 M, 13.7%) | 1,636 (3.8 M, 17.6%) |
| Non-Hispanic White | 22,959 (141 M, 68.3 %) | 2,270 (12 M, 55.8%) |
| Other | 4,850 (14 M, 6.9%) | 834 (2.4 M, 11.1%) |
| Educational Attainment | | |
| Less than 9^th^ Grade | 6,093 (12 M, 5.7%) | 1,030 (2.0 M, 9.6%) |
| 9^th^ – 11^th^ Grade | 7,696 (23 M, 11.1%) | 1,047 (2.8 M, 13.2%) |
| High School Graduate or Equivalent | 12,182 (50 M, 24.2%) | 1,464 (5.3 M, 25.0%) |
| Some College or Associates Degree | 14,990 (64 M, 30.9%) | 1,597 (5.8 M, 27.5%) |
| College Graduate or Above | 11,400 (58 M, 28.1%) | 1,182 (5.2 M, 24.7%) |
| Smoking History | | |
| Never Smoking | 28,536 (111 M, 54.5%) | 3,647 (12 M, 56.2%) |
| Former Smoking | 12,929 (51 M, 24.7%) | 1,431 (4.7 M, 22.1%) |
| Current Smoking | 10,873 (44 M, 20.8%) | 1,265 (4.6 M, 21.7%) |
| Obesity | | |
| BMI 18.5-24.9 | 14,400 (60 M, 29.9%) | 1,738 (6.0 M, 29.1%) |
| BMI 25-29.9 | 17,373 (68 M, 33.8%) | 2,068 (6.9 M, 33.7%) |
| BMI ≥ 30 | 19,012 (73 M, 36.3%) | 2,315 (7.6 M, 37.2%) |
| Diabetes Mellitus | 8,642 (25 M, 12.2%) | 1,184 (3.1 M, 14.6%) |
| Dyslipidemia | 24,546 (99 M, 47.6%) | 2,445 (8.1 M, 38.1%) |
| Prior Cardiovascular Disease | 4,438 (13 M, 6.5%) | 566 (1.5 M, 6.9%) |

BMI, body mass index.

**Supplemental Table 2. Adjusted hazard ratios for cardiovascular and all-cause mortality by age group and hypertension subtype for US adults with and without diabetes mellitus, NHANES 1999–2020.**

|  | Cardiovascular Mortality | | All-Cause Mortality | |
| --- | --- | --- | --- | --- |
|  | Without DM | With DM | Without DM | With DM |
| Normotension | | | | |
| < 65 years | 1.00 | 1.00 | 1.00 | 1.00 |
| ≥ 65 years | 1.00 | 1.00 | 1.00 | 1.00 |
| Isolated Systolic Hypertension | | | | |
| < 65 years | 1.68  (1.11-2.56) | 2.37  (1.35-4.17) | 1.28  (1.07-1.54) | 2.35  (1.79-3.10) |
| ≥ 65 years | 1.12  (1.04-1.25) | 1.70  (1.43-2.01) | 1.15  (1.05-1.30) | 1.43  (1.27-1.61) |
| Isolated Diastolic Hypertension | | | | |
| < 65 years | 0.94  (0.52-1.71) | 5.16  (2.44-10.95) | 0.90  (0.68-1.18) | 2.45  (1.52-3.95) |
| ≥ 65 years | 0.44  (0.17-1.09) | 0.96  (0.11-7.99) | 0.86  (0.58-1.28) | 0.97  (0.49-2.36) |
| Systolic-Diastolic Hypertension | | | | |
| < 65 years | 1.73  (1.19-2.51) | 2.65  (1.63-4.31) | 1.34  (1.10-1.64) | 2.05  (1.57-2.69) |
| ≥ 65 years | 1.21  (1.03-1.45) | 2.13  (1.43-3.16) | 1.15  (1.05-1.28) | 1.55  (1.16-2.05) |
| HTN/DM Interaction | | | | |
| < 65 years | p <0.01 | | p <0.01 | |
| ≥ 65 years | p <0.01 | | p <0.01 | |

Separate regression analyses performed for individuals < 65 years and ≥ 65 years, adjusting for age, ethnicity, gender, educational attainment, obesity, tobacco use, dyslipidemia, use of antihypertensive agents, and prior cardiovascular disease. HTN, hypertension; DM, diabetes mellitus.

**Supplemental Table 3. Adjusted hazard ratios for cardiovascular and all-cause mortality by hypertension subtype for US adults with and without diabetes mellitus using JNC7 hypertension cutoffs of 140/90 mmHg, NHANES 1999–2020.**

|  | Cardiovascular Mortality | | All-Cause Mortality | |
| --- | --- | --- | --- | --- |
|  | Without DM | With DM | Without DM | With DM |
| Normotension | 1.00 | 1.00 | 1.00 | 1.00 |
| Isolated Systolic Hypertension | 1.19  (1.01-1.41) | 1.91  (1.55-2.37) | 1.18  (1.09-1.29) | 1.54  (1.37-1.74) |
| Isolated Diastolic Hypertension | 0.77  (0.51-1.64) | 3.82  (1.16-12.56) | 1.12  (0.76-1.67) | 2.33  (1.07-5.62) |
| Systolic-Diastolic Hypertension | 1.51  (1.11-2.06) | 1.75  (1.14 – 3.26) | 1.29  (1.10-1.51) | 1.61  (1.07-2.42) |
| HTN/DM Interaction | p <0.01 | | p <0.01 | |

All analyses adjusted for age, ethnicity, gender, educational attainment, obesity, tobacco use, dyslipidemia, use of antihypertensive agents, and prior cardiovascular disease. HTN, hypertension; DM, diabetes mellitus.
